# Supplementary material for: Assay to visualize specific protein oxidation reveals spatio-temporal regulation of SHP2
Source: Nat Commun. 2017 Sep 6;8:466. doi: 10.1038/s41467-017-00503-w (PMC5587708; doi:10.1038/s41467-017-00503-w)
Supplement: Supplementary file 1 — Supplementary Information [file 41467_2017_503_MOESM1_ESM.pdf]

File Name: Supplementary Information
Description: Supplementary Figures

File Name: Peer Review File

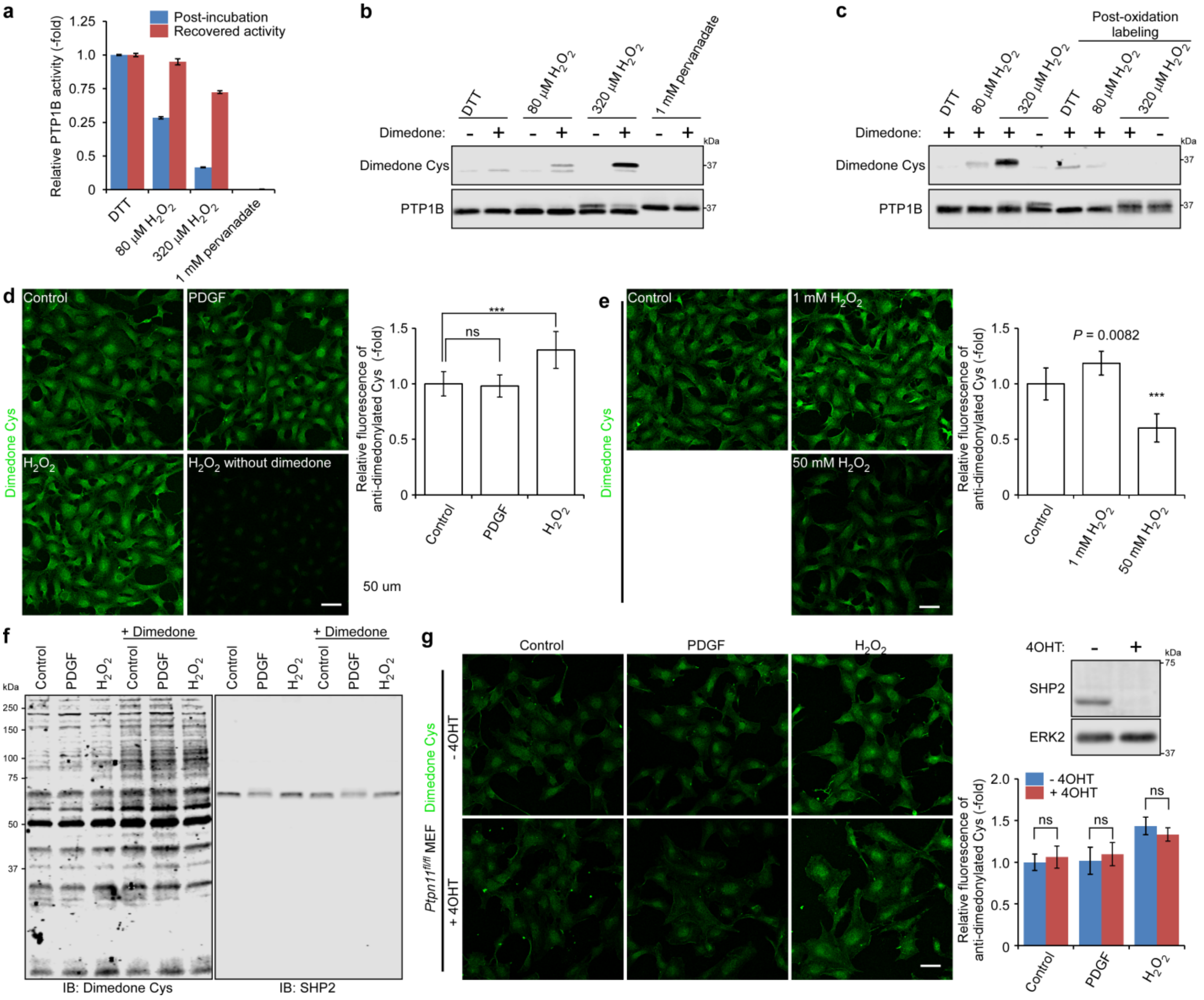

Supplementary Fig. 1. Dimedone labels reversibly oxidized Cys residues *in vitro* and in cells. (**a**) Purified recombinant PTP1B (1-321) was incubated under the indicated conditions for 30 min. Reaction mixtures were then subjected to *in vitro* phosphatase assays using *p*NPP as substrate, either immediately (post-oxidation) or after an additional incubation with DTT (10 mM) and catalase (5 U) (recovered activity). Relative phosphatase activities are shown in the graph, setting the activity of DTT-treated PTP1B as 1. Representative data from 2 experiments are shown. Error bars indicate SD (n = 3 technical replicates). (**b**) PTP1B was incubated with or without 5 mM dimedone under the indicated conditions for 5 min, followed by SDS-PAGE and immunoblotting with anti-dimedone Cys and anti-PTP1B antibodies. Representative data from 3 experiments are shown. (**c**) Purified PTP1B was labeled with dimedone (5 mM) for 5 min under the indicated conditions or after pre-incubation with the indicated concentrations of

DTT or H_2O_2, followed by immunoblotting with anti-dimedone-Cys and anti-PTP1B antibodies. Representative data from 2 experiments are shown. (**d**) Serum-starved Swiss 3T3 cells were treated with PDGF-BB (50 ng ml^{-1}) or H_2O_2 (1 mM) for 10 min. Cells were fixed in the presence or absence of dimedone (5 mM) for 5 min, and subjected to immunostaining with anti-dimedone-Cys antibodies (green). Representative images are shown for each condition from one of 2 independent experiments. The graph shows the average intensities of the anti-dimedone Cys signal subtracted by background signal in the images, relative to the values in untreated control cells (n = 10 images). Error bars represent SD. ***P<0.0001, ns: not significant, ANOVA with Bonferroni/Dunn's post-hoc test. (**e**) Serum-starved Swiss 3T3 cells were treated with H_2O_2 (1 mM or 50 mM) for 10 min. Cells were then fixed in the presence of dimedone (5 mM) for 5 min, and subjected to immunostaining with anti-dimedone Cys antibodies (green). Representative images are shown for each condition from one of 2 independent experiments. The graph shows the average intensities of the anti-dimedone Cys signal with the background signal in each image subtracted, relative to the values in untreated control cells (n = 10 images). Error bars represent SD. ***P<0.0001 to control cells, ANOVA with Bonferroni/Dunn's post-hoc test. (**f**) Serum-starved Swiss 3T3 cells were treated with PDGF-BB (50 ng ml^{-1}) or H_2O_2 (1 mM) for 10 min, followed by treatment with dimedone (5 mM) for 30 min. Lysates were immunoblotted with anti-dimedone-Cys and anti-SHP2 antibodies. Representative images are shown from one of 2 independent experiments. (**g**) Serum-starved *Ptpn11*$^{fl/fl}$ MEFs expressing CRE-ER^{Tam} treated with or without 4-hydroxytamoxifen (4OHT) were stimulated with PDGF-BB (50 ng ml^{-1}) or H_2O_2 (1 mM) for 10 min. Cells were fixed in the presence of dimedone, and immunostained with anti-dimedone Cys antibodies (green). Representative images are shown for each condition from one of 2 independent experiments. Lysates from *Ptpn11*$^{fl/fl}$ MEFs were immunoblotted with anti-SHP2 and anti-ERK2 antibodies. The graph shows the average intensities of the anti-dimedonylated cysteine signal, with the background signal in each image subtracted, relative to the values in untreated cells (n = 10 images). Error bars represent SD. ns: not significant, ANOVA with Bonferroni/Dunn's post-hoc test. Scale bars: 50 μm.

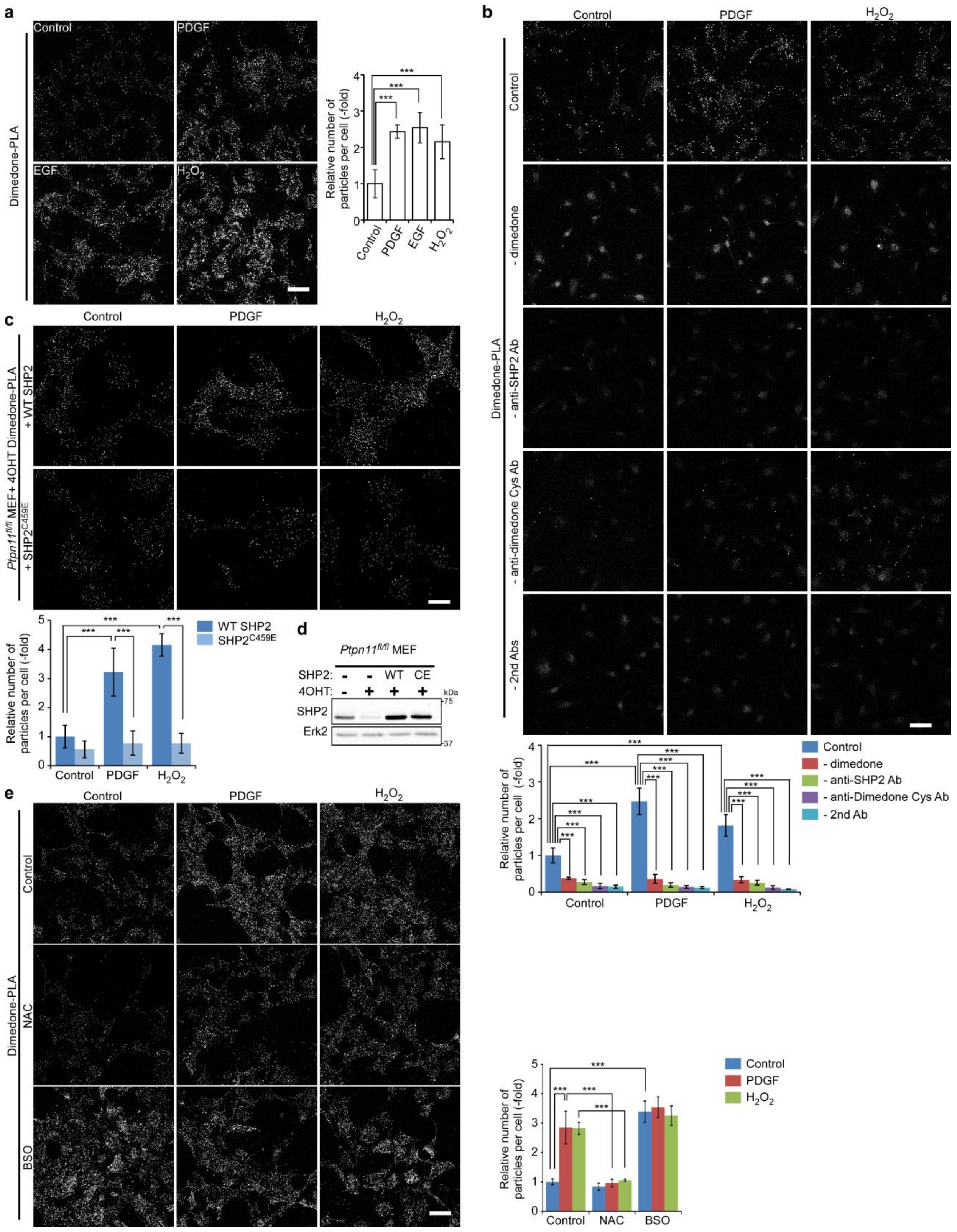
a
Control
PDGF
EGF
H_2O_2
Dimedone-PLA
Relative number of particles per cell (-fold)
***
b
- dimedone
- anti-SHP2 Ab
- anti-dimedone Cys Ab
- 2nd Abs
- anti-Dimedone Cys Ab
- 2nd Ab
c
$Ptpn11^{fl/fl}$ MEF+ 4OHT Dimedone-PLA
+ WT SHP2
+ $SHP2^{C459E}$
WT SHP2
$SHP2^{C459E}$
d
$Ptpn11^{fl/fl}$ MEF
SHP2:
4OHT:
WT
CE
kDa
75
37
SHP2
Erk2
e
NAC
BSO

Supplementary Fig. 2. Specific detection of oxidized SHP2 by dimedone-PLA. (**a**) Serum-starved Swiss 3T3 cells were stimulated with PDGF-BB (50 ng ml^{-1}), EGF (50 ng ml^{-1}), or H_2O_2 (1 mM) for 10 min, fixed in the presence of dimedone (5 mM) for 5 min, and subjected to dimedone-PLA (gray). Representative images are shown for each condition from one of 3 independent experiments. The graph shows the average number of PLA signals per cell (*n* = 6 images for each condition, 5-20 cells in an image), relative to unstimulated control cells (normalized to 1). Error bars represent SD. ***P<0.0001, ANOVA with Bonferroni/Dunn's post-hoc test. (**b**) Serum-starved Swiss 3T3 cells were stimulated with PDGF-BB (50 ng ml^{-1}) or H_2O_2 (1 mM) for 10 min, fixed in the presence or absence of dimedone for 5 min, and subjected to dimedone-PLA (gray) with or without either primary or secondary antibody. Representative images are shown for each condition from one of 2 independent experiments. The graph shows the average number of PLA signals per cell (*n* = 6 images for each condition, 5-20 cells in an image) relative to control cells without stimulation. Error bars represent SD. ***P<0.0001, ANOVA with Bonferroni/Dunn's post-hoc test. (**c**) 4-OHT-treated, serum-starved $Ptpn11^{fl/fl}$ MEFs expressing Cre-ER^{Tam} with re-expression of either wild-type SHP2 (WT SHP2) or C459E SHP2 ($SHP2^{C459E}$) were stimulated with PDGF-BB (50 ng ml^{-1}) or H_2O_2 (1 mM) for 10 min. Cells were fixed in the presence of dimedone, and subjected to dimedone-PLA (gray). Representative images are shown for each condition from one of 2 independent experiments. The graph shows the average number of PLA signals per cell (*n* = 6 images for each condition, 5-20 cells in an image) relative to control cells without stimulation. Error bars represent SD. ***P<0.0001, ANOVA with Bonferroni/Dunn's post-hoc test. (**d**) Lysates from $Ptpn11^{fl/fl}$ MEFs with or without 4-OHT treatment, and MEFs treated with 4-OHT re-expressing either WT SHP2 or $SHP2^{C459E}$ were immunoblotted with indicated antibodies. Representative immunoblots are shown from one of 2 experiments. (**e**) Serum-starved Swiss 3T3 cells, pre-treated with NAC (10 mM, 15 min) or BSO (2.5 mM, 16 h), were stimulated with PDGF-BB (50 ng ml^{-1}) or H_2O_2 (1 mM) for 10 min, fixed in the presence of dimedone for 5 min, and subjected to dimedone-PLA (gray). Representative images are shown for each condition from one of 2 independent experiments. The graph shows the average number of PLA signals per cell (*n* = 6 images for each condition, 5-20 cells in an image) relative to unstimulated control cells. Error bars represent SD. ***P<0.0001, ANOVA with Bonferroni/Dunn's post-hoc test. Scale bars: 50 μm.

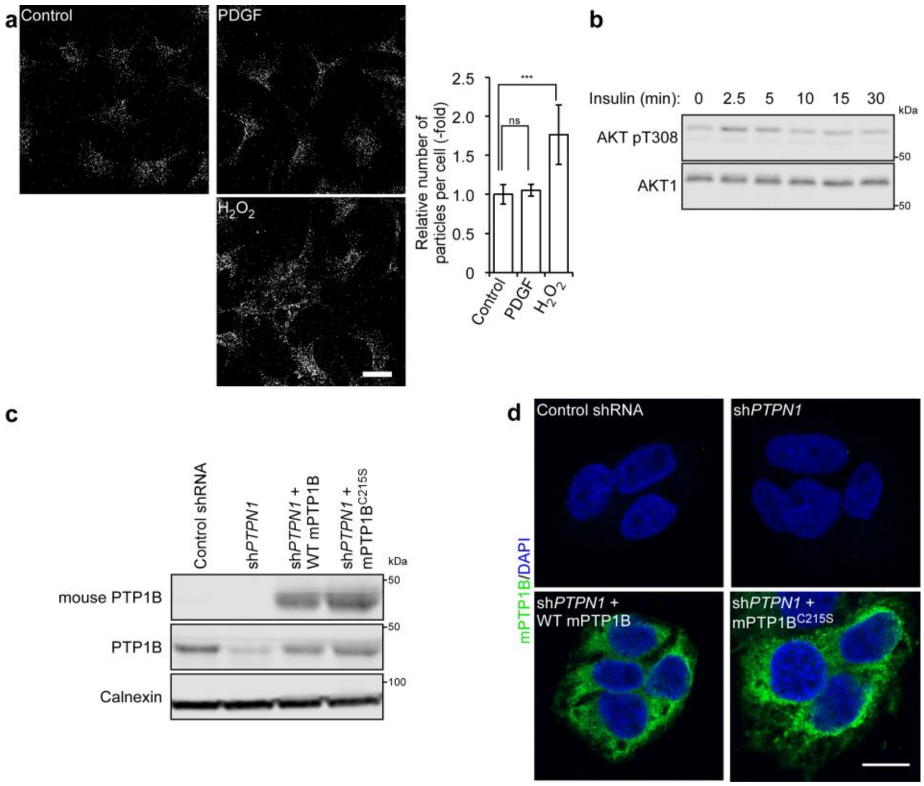

Supplementary Fig. 3. Dimedone-PLA detects oxidized PTP1B. (**a**) Serum-starved Swiss 3T3 cells were treated with PDGF-BB (50 ng ml^{-1}) or H_2O_2 (1 mM) for 10 min, fixed in the presence of dimedone (5 mM) for 5 min, and subjected to dimedone-PLA for PTP1B (gray). Representative images are shown for each condition from one of 2 independent experiments. The graph shows the average number of PLA signals per cell (n = 6 images for each condition, 5-20 cells in an image), relative to untreated control cells (set to 1). Error bars represent SD. ***P<0.0001, ns: not significant, ANOVA with Bonferroni/Dunn's post-hoc test. Scale bar: 50 μm. (**b**) Serum-starved HepG2 cells were treated with insulin (25 nM) for the indicated times. Lysates were immunoblotted with anti-AKT pT308 and anti-AKT1 antibodies. Representative images are shown for each condition from one of 2 independent experiments. (**c**) Lysates from HepG2 cells expressing control shRNA or shRNA targeting human *PTPN1* (sh*PTPN1*) with or without stable expression of mouse PTP1B (WT mPTP1B) or C215S PTP1B (mPTP1B^{C215S}) were immunoblotted with anti-mouse PTP1B, anti-PTP1B antibodies that recognize both mouse and human PTP1B, and anti-calnexin antibodies. Representative immunoblots are shown from one of 2 independent experiments. (**d**) HepG2 cells expressing control shRNA or sh*PTPN1* with or without stable expression of WT mPTP1B or mPTP1B^{C215S} were immunostained with anti-mouse PTP1B antibodies (green). Nuclei were visualized with DAPI (blue). Representative images are shown for each condition from one of 2 independent experiments. Scale bar: 10 μm.

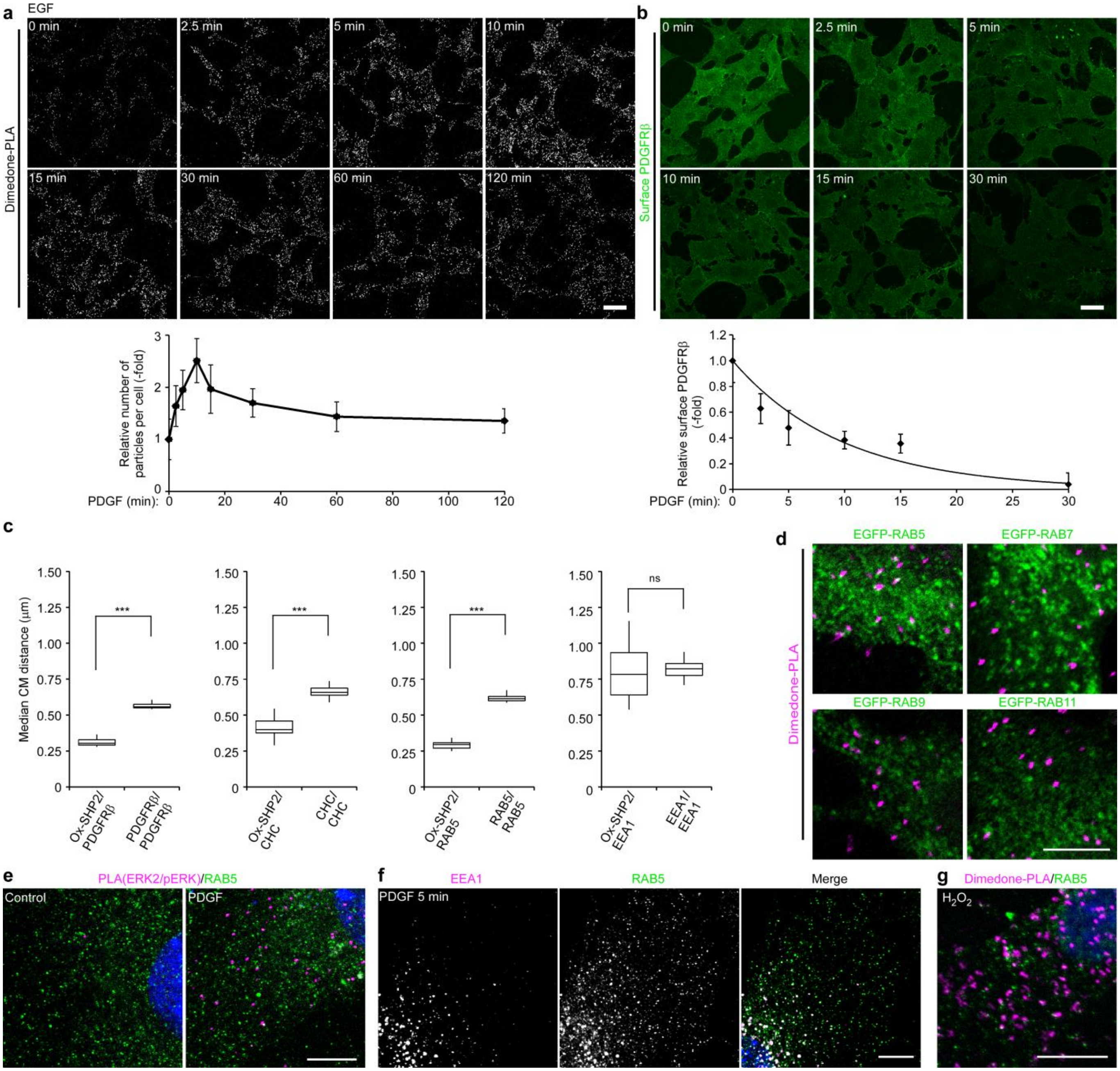

Supplementary Fig. 4. Spatio-temporal dynamics of SHP2 oxidation. (**a**) Serum-starved Swiss 3T3 cells were stimulated with EGF (50 ng ml^{-1}) for the indicated times, fixed in the presence of dimedone, and subjected to dimedone-PLA. Representative images are shown for each condition from one of 2 independent experiments. The graph shows the average number of PLA signals per cell (*n* = 6 images for each condition, 5-20 cells in an image) relative to unstimulated control cells (normalized to 1). Error bars represent SD. Scale bar: 50 μm. (**b**) Serum-starved Swiss 3T3 cells were stimulated with PDGF (50 ng ml^{-1}) for the indicated times, fixed, and subjected to immunostaining with anti-PDGFRβ antibodies

without permeabilization to visualize surface PDGFRβ. Representative images are shown for each condition from one of 2 independent experiments. The graph shows the average fluorescence intensities of the surface PDGFRβ signal with the background signal in the image subtracted, relative to the values in untreated cells (*n* = 10 images). Error bars represent SD. Scale bar: 50 μm. (**c**) Serum-starved Swiss 3T3 cells were treated with 50 ng ml^{-1} PDGF-BB for 2.5 min (see Fig. 2b). Median distances of the centers of mass between punctate signals of ox-SHP2 and of the nearest indicated marker signal, or median distances of between punctate signals of indicated marker themselves, were obtained by object-based image analysis. Box-whisker plots show the 25th-75th percentiles (boxes) and the 5th-95th percentiles (whiskers) of median distances (*n* = 50 cells). ***P<0.0001, ns: not significant, unpaired *t* test (PDGFRβ and RAB5) or unpaired Welch's *t* test (CHC and EEA1), as appropriate. (**d**) Swiss 3T3 cells expressing EGFP-fused RAB5, RAB7, RAB9, or RAB11 were serum-starved, stimulated with PDGF-BB (50 ng ml^{-1}) for 10 min, and then fixed in the presence of dimedone. Dimedone-PLA (magenta) and anti-GFP staining (green) are shown. Representative images are shown for each condition from one of 2 independent experiments. Scale bar: 10 μm. (**e**) Serum-starved Swiss 3T3 cells were stimulated with PDGF-BB (50 ng ml^{-1}) for 5 min, fixed, subjected to PLA with anti-ERK2 and anti-pERK1/2 antibodies (magenta), and co-stained with the anti-RAB5 antibody (green). Representative images are shown for each condition from one of 2 independent experiments. Scale bar: 10 μm. (**f**) Serum-starved Swiss 3T3 cells were treated with PDGF-BB (50 ng ml^{-1}) for 5 min and immunostained with anti-RAB5 (gray or green) and anti-EEA1 (gray or magenta) antibodies. Nuclei were labeled with DAPI (blue). Representative images are shown from a single experiment. Scale bar: 10 μm. (**g**) Serum-starved Swiss 3T3 cells were treated with H_2O_2 (1 mM) for 10 min and subjected to dimedone-PLA (magenta), followed by immunostaining with anti-RAB5 antibody. Nuclei were labeled with DAPI (blue). Representative images are shown from one of 2 independent experiments. Scale bar: 10 μm.

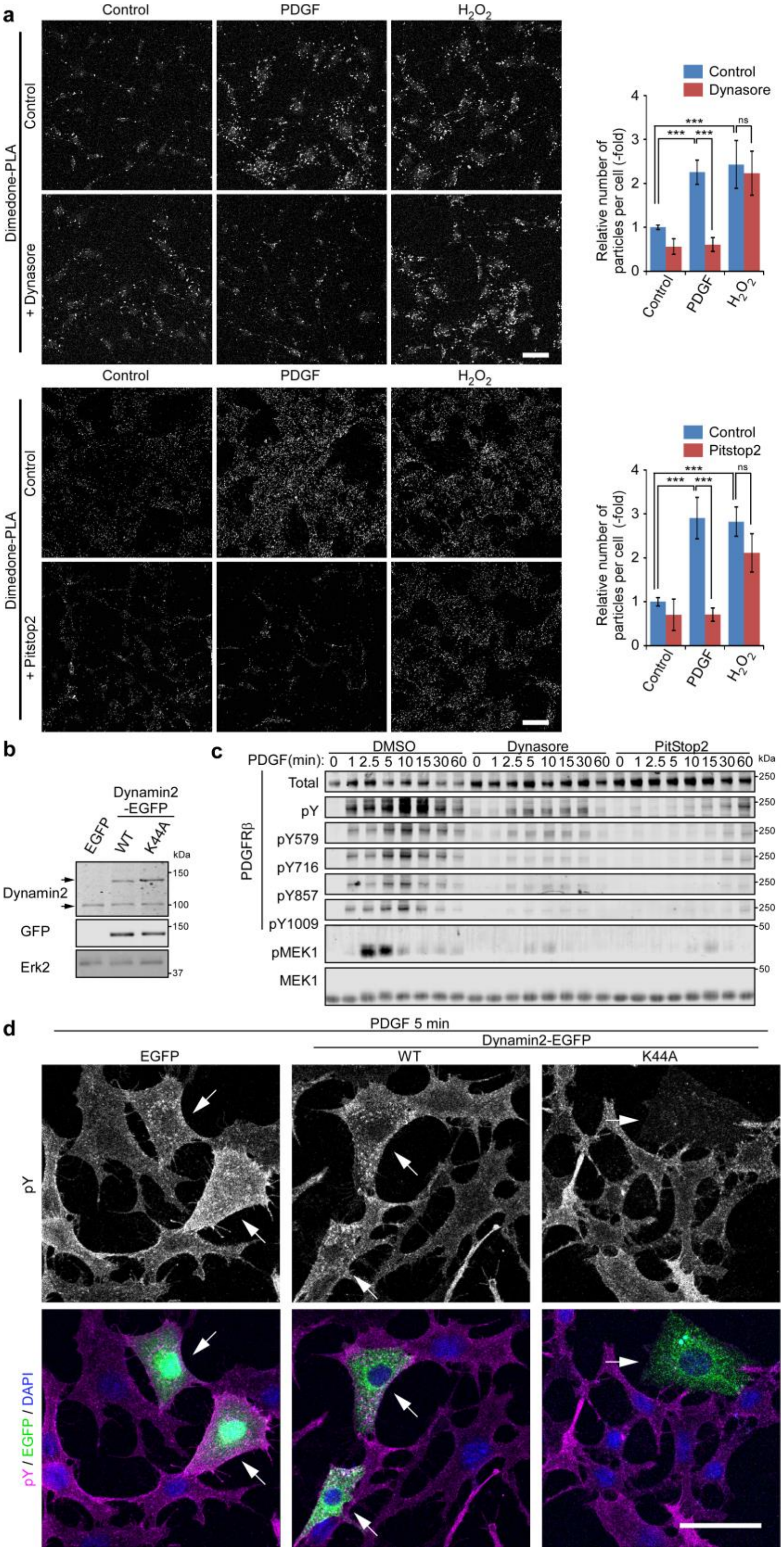
a
Control
PDGF
H_2O_2
Dimedone-PLA
Control
+ Dynasore
Control
Dynasore
Relative number of particles per cell (-fold)
ns
PDGF
+ Pitstop2
Pitstop2
b
Dynamin2 -EGFP
EGFP
WT
K44A
kDa
Dynamin2
GFP
Erk2
c
DMSO
Dynasore
PitStop2
PDGF(min):
PDGFRβ
Total
pY
pY579
pY716
pY857
pY1009
pMEK1
MEK1
d
PDGF 5 min
Dynamin2-EGFP
pY
pY / EGFP / DAPI

Supplementary Fig. 5. Involvement of endocytosis in SHP2 oxidation. (**a**) Serum-starved Swiss 3T3 cells were pre-treated with Dynasore (50 μM) (top panels) or Pitstop® 2 (25 μM) (bottom panels) for 30 min, stimulated with PDGF-BB (50 ng ml^{-1}) or H_2O_2 (1 mM) for 10 min in the presence of the inhibitor, and then subjected to dimedone-PLA (gray). Representative images are shown for each condition from one of 3 independent experiments. Graphs show the average number of PLA signals per cell (n = 6 images for each condition, 5-20 cells in an image), relative to unstimulated control cells (set to 1). Error bars represent SD. ***P<0.0001, ns: not significant, ANOVA with Bonferroni/Dunn's post-hoc test. (**b**) Lysates from Swiss 3T3 cells expressing EGFP-fused wild-type dynamin 2 (WT Dynamin2) or dominant-negative dynamin 2 ($dynamin2^{K44A}$) (see Fig. 3d) were immunoblotted with the indicated antibodies. Arrows indicate exogenous (EGFP-fused) and endogenous dynamin, respectively. Representative immunoblots are shown from one of 2 experiments. (**c**) Serum-starved Swiss 3T3 cells were pre-treated with Dynasore (50 μM) or Pitstop® 2 (25 μM) for 30 min, and stimulated with PDGF-BB (50 ng ml^{-1}) for the indicated times. Lysates were subjected to immunoblotting with the indicated antibodies. Representative immunoblots from one of 3 experiments are shown. (**d**) Serum-starved Swiss 3T3 cells expressing EGFP, EGFP-WT dynamin2 or EGFP-K44A dynamin2 were stimulated with PDGF-BB (50 ng ml^{-1}) for 5 min. Cells were stained with an anti-pTyr (gray or magenta) antibody cocktail. EGFP is shown in green; nuclei are stained with DAPI. Representative images are shown for each condition from one of 2 independent experiments. Arrows indicate cells expressing EGFP or EGFP-dynamin2. Scale bars: 50 μm.

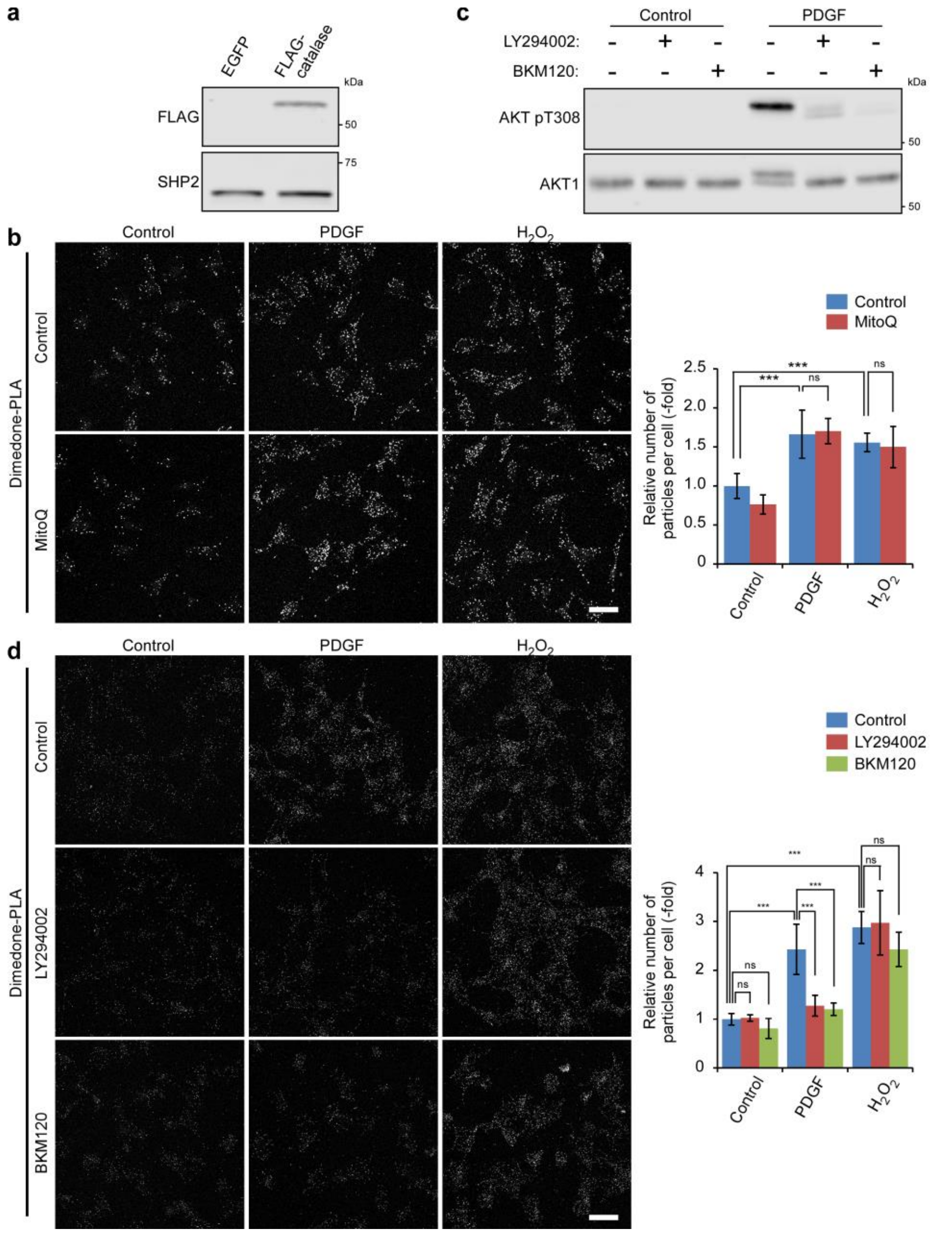

Supplementary Fig. 6. PDGF-evoked SHP2 oxidation requires H_2O_2 and PI3K activity but not mitochondrial ROS. (**a**) Lysates from Swiss 3T3 cells stably expressing EGFP or Flag-tagged cytoplasmic catalase were immunoblotted with the indicated antibodies (see Fig. 4a). Representative immunoblots from one of 2 experiments are shown. (**b**) Serum-starved Swiss 3T3 cells were pre-treated with MitoQ (1 μM) for 1 h, stimulated with PDGF-BB (50 ng ml^{-1}) or H_2O_2 (1 mM) for 10 min, fixed in the presence of dimedone, and subjected to dimedone-PLA (gray). Representative images are shown for each condition from one of 2 independent experiments. Graphs show the average number of PLA signals per cell (n = 6 images for each condition, 5-15 cells in an image), relative to unstumulated cells. Error bars represent SD.

***P<0.0001, ns: not significant, ANOVA with Bonferroni/Dunn's post-hoc test. (**c**) Serum-starved Swiss 3T3 cells were pre-treated with LY294002 (20 μM) or BKM120 (20 μM) for 1 h, stimulated with PDGF-BB (50 ng ml^{-1}) for 10 min. Lysates were subjected to immunoblotting with anti-AKT pT308 and anti-AKT antibodies. Representative immunoblots from one of 2 experiments are shown. (**d**) Serum-starved Swiss 3T3 cells were pre-treated with LY294002 (20 μM) or BKM120 (20 μM) for 1 h, stimulated with PDGF-BB (50 ng ml^{-1}) or H_2O_2 (1 mM), fixed in the presence of dimedone, and subjected to dimedone-PLA (gray). Representative images are shown for each condition from one of 2 independent experiments. The graph shows the average number of PLA signals per cell (n = 6 images for each condition, 10-25 cells in an image), relative to unstimulated cells. Error bars represent SD. ***P<0.0001, ns: not significant, ANOVA with Bonferroni/Dunn's post-hoc test. Scale bars: 50 μm.

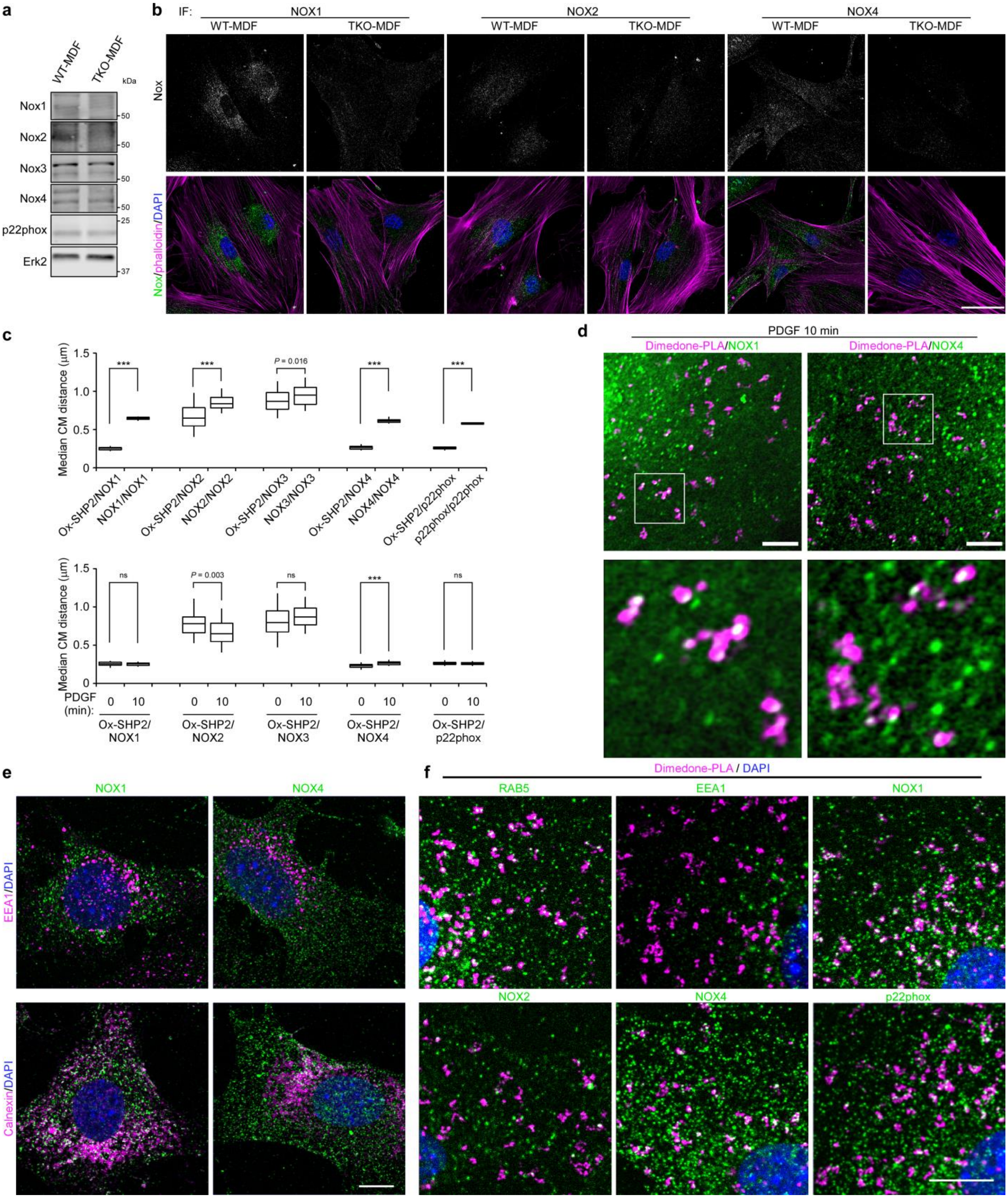
a
WT-MDF
TKO-MDF
kDa
Nox1
Nox2
Nox3
Nox4
p22phox
Erk2
50
50
50
50
25
37
b
IF:
NOX1
NOX2
NOX4
WT-MDF
TKO-MDF
Nox
Nox/phalloidin/DAPI
c
Median CM distance (μm)
***
P = 0.016
ns
P = 0.003
Ox-SHP2/NOX1
NOX1/NOX1
Ox-SHP2/NOX2
NOX2/NOX2
Ox-SHP2/NOX3
NOX3/NOX3
Ox-SHP2/NOX4
NOX4/NOX4
Ox-SHP2/p22phox
p22phox/p22phox
PDGF (min):
Ox-SHP2/ NOX1
Ox-SHP2/ NOX2
Ox-SHP2/ NOX3
Ox-SHP2/ NOX4
Ox-SHP2/ p22phox
d
PDGF 10 min
Dimedone-PLA/NOX1
Dimedone-PLA/NOX4
e
NOX1
NOX4
EEA1/DAPI
Calnexin/DAPI
f
Dimedone-PLA / DAPI
RAB5
EEA1
NOX1
NOX2
NOX4
p22phox

Supplementary Fig. 7. NOX complexes co-localize with oxidized SHP2. (**a**) Anti-NOX antibodies are specific. Lysates from primary murine dermal fibroblasts (MDFs) from wild type (WT) or *Nox1,2,4* triple-KO (TKO) animals were subjected to immunoblotting with the indicated antibodies. Representative immunoblots are shown from one of 2 experiments. (**b**) WT- or TKO-MDFs were immunostained with the indicated anti-NOX antibodies (gray and green). Nuclei and actin cytoskeleton were stained with DAPI (blue) and phalloidin (magenta), respectively. Representative images are shown for each condition from one of 2 independent experiments. Scale bar: 50 μm. (**c**) Serum-starved Swiss 3T3 cells were treated with PDGF-BB (50 ng ml^{-1}) for 10 min. The median distances of centers of mass between punctate signals of ox-SHP2 and the nearest indicated marker signal, or median distances of between punctate signals of the indicated markers themselves, were obtained by object-based image analysis. Also see Fig. 3c. Top panel shows comparisons between ox-SHP2-to-marker and marker-to marker distances at 10 min after PDGF stimulation. Note that although all of the inter-marker distances are less than the intra-marker distances, the inter-marker distances between the ox-SHP2/NOX2 and ox-SHP2/NOX3 comparisons are far too large to indicate co-localization (compare distance scale on y-axis). Bottom panel shows data from the same experiment indicating ox-SHP2-to-marker distances before and after PDGF-BB (10 min) stimulation. Box-whisker plots with the 25th-75th percentiles (boxes) and the 5th-95th percentiles (whiskers) of the median distances (n = 50 cells each), ***P<0.0001, unpaired t test or unpaired Welch's t test, as appropriate. (**d**) Serum-starved Swiss 3T3 cells were stimulated with PDGF-BB (50 ng ml^{-1}) for 10 min, and subjected to dimedone-PLA labeling of ox-SHP2 (magenta) followed by immunolabeling with either anti-NOX1 or anti-NOX4 antibodies (green). Representative semi-super resolution microscopic images (AiryScan) are shown from one of 2 independent experiments. Higher magnification images of the boxed regions are shown at the bottom. Scale bars: 5 μm. (**e**) Swiss 3T3 cells were immunostained with anti-NOX1 or anti-NOX4 (green) and anti-EEA1 or anti-calnexin (ER marker) (magenta) antibodies. Nuclei were stained with DAPI (blue). Representative images for each condition from one of 2 independent experiments are shown. Scale bar: 10 μm. (**f**) Serum-starved Swiss 3T3 fibroblasts were stimulated with EGF (50 ng ml^{-1}) for 5 min, and fixed in the presence of dimedone. Co-staining of dimedone-PLA labeling ox-SHP2 (magenta) and indicated antibodies (green) is shown. Representative images are shown for each condition from one of 2 independent experiments. Nuclei were stained with DAPI (blue). Scale bar: 10 μm.

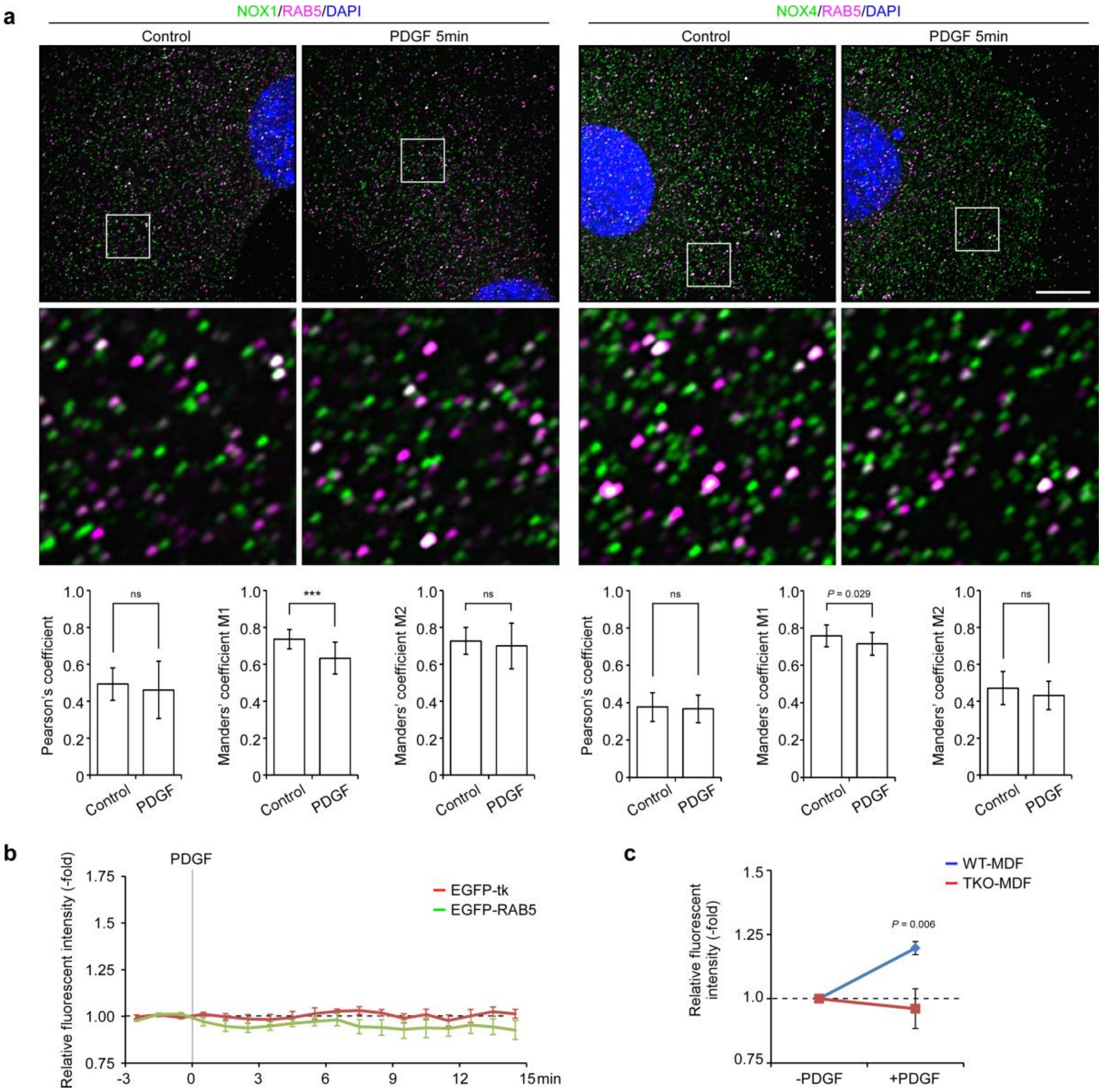

Supplementary Fig. 8. PDGF-evoked redoxosome formation. (**a**) Serum-starved Swiss 3T3 cells were stimulated with or without PDGF-BB (50 ng ml^{-1}) for 5 min, immunostained with anti-RAB5 (magenta) and anti-NOX4 (green) antibodies and visualized by semi-super resolution microscopy (AiryScan). Higher magnification images of the boxed regions are shown at the bottom. Representative images are shown for each condition from one of 2 independent experiments. Scale bar: 10 μm. Graphs show Pearson's or Manders' coefficients for co-localization of RAB5 and NOX1 (left) or RAB5 and NOX4 (right) before and after PDGF-BB stimulation (n = 20 ROIs from 10 cells). ***P<0.0001, ns: not significant, Unpaired two-tailed t test or Welch's t test, as appropriate. Error bars represent SD. (**b**) Serum-starved Swiss 3T3 cells expressing plasma membrane-targeted EGFP (EGFP-tk) or RAB5-fused EGFP (EGFP-RAB5) were subjected to live-cell time-lapse imaging, and fluorescence intensity was

monitored at 1 min intervals. PDGF-BB (50 ng ml^{-1}) was added, as indicated, after the third scan. Graph shows the relative fluorescence intensities of individual cells (n = 7 cells each from individual time-lapse experiments), setting the average of the first 3 time-points to 1. Error bars represent SEM. (**c**) Serum-starved WT- or TKO-MDFs expressing HyPer3-RAB5 were subjected to live-cell time-lapse imaging, and fluorescence intensity was monitored. Graph shows the relative fluorescence intensities of individual cells (n = 5 cells each from individual time-lapse experiments) before and 5 min after PDGF-BB stimulation, with unstimulated cell values normalized to 1. P value was calculated with two-tailed Welch's *t* test. Error bars represent SD.

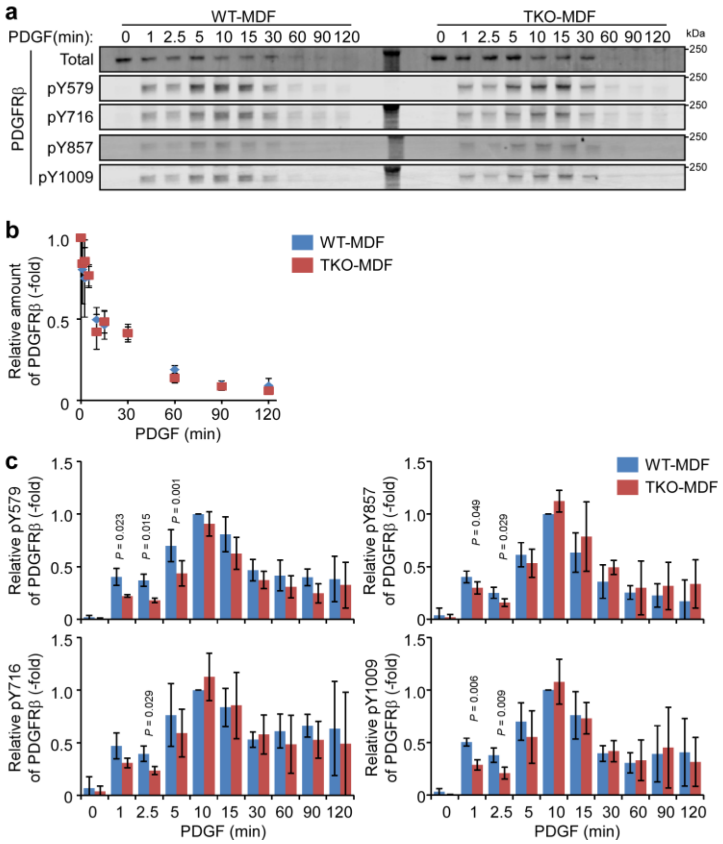

Supplementary Fig. 9. NOX activity is necessary for PDGF signaling. (**a**) Serum-starved WT- or TKO-MDFs were stimulated with PDGF-BB (50 ng ml^{-1}) for the indicated times. Lysates were subjected to immunoblotting with the indicated antibodies. Representative immunoblots are shown from one of 4 experiments. (**b**) Graph shows the relative amount of PDGFRβ (compared to ERK2) at various times following PDGF addition, as obtained from immunoblots, with the level at time 0 normalized to 1. Data are means ±SD (n = 4). (**c**) Relative phosphorylation of PDGFRβ on the indicated tyrosine residues compared to total PDGFRβ at the indicated times, as obtained from immunoblots. For each residue, the value in WT-MDF at 10 min is set as 1. Data are means ±SD (n = 4). P values were calculated using paired two-tailed t tests.

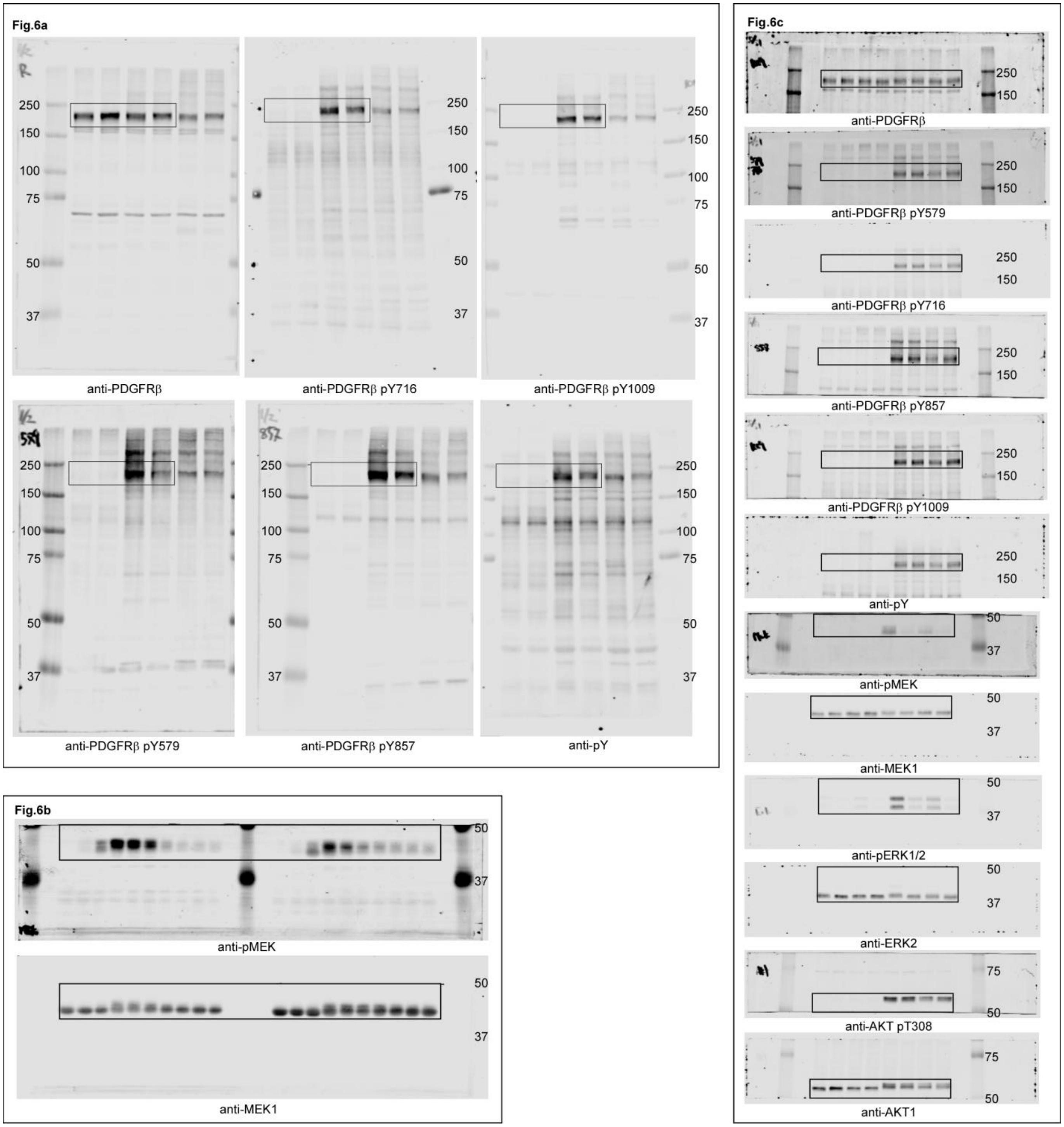

Supplementary Fig. 10. Uncropped immunoblot images presented in Figures.
